# Surface Roughness Evaluation in Thin EN AW-6086-T6 Alloy Plates after Face Milling Process with Different Strategies

**DOI:** 10.3390/ma14113036

**Published:** 2021-06-02

**Authors:** Daniel Chuchala, Michal Dobrzynski, Danil Yurievich Pimenov, Kazimierz A. Orlowski, Grzegorz Krolczyk, Khaled Giasin

**Affiliations:** 1 Faculty of Mechanical Engineering and Ship Technology, Gdańsk University of Technology, 80-233 Gdańsk, Poland; michal.dobrzynski@pg.edu.pl (M.D.); kazimierz.orlowski@pg.edu.pl (K.A.O.); 2 Department of Automated Mechanical Engineering, South Ural State University, Lenin Prosp. 76, 454080 Chelyabinsk, Russia; danil_u@rambler.ru; 3 Department of Manufacturing Engineering and Automation Products, Opole University of Technology, 45-758 Opole, Poland; g.krolczyk@po.opole.pl; 4 School of Mechanical and Design Engineering, University of Portsmouth, Portsmouth PO1 3DJ, UK; khaled.giasin@port.ac.uk

**Keywords:** face milling, milling strategy, surface roughness, aluminium alloy, rolling direction, residual stresses

## Abstract

Lightweight alloys made from aluminium are used to manufacture cars, trains and planes. The main parts most often manufactured from thin sheets requiring the use of milling in the manufacturing process are front panels for control systems, housing parts for electrical and electronic components. As a result of the final phase of the manufacturing process, cold rolling, residual stresses remain in the surface layers, which can influence the cutting processes carried out on these materials. The main aim of this study was to verify whether the strategy of removing the outer material layers of aluminium alloy sheets affects the surface roughness after the face milling process. EN AW-6082-T6 aluminium alloy thin plates with three different thicknesses and with two directions relative to the cold rolling process direction (longitudinal and transverse) were analysed. Three different strategies for removing the outer layers of the material by face milling were considered. Noticeable differences in surface roughness 2D and 3D parameters were found among all machining strategies and for both rolling directions, but these differences were not statistically significant. The lowest values of *Ra* = 0.34 µm were measured for the S#3 strategy, which asymmetrically removed material from both sides of the plate (main and back), for an 8-mm-thick plate in the transverse rolling direction. The highest values of *Ra* = 0.48 µm were measured for a 6-mm-thick plate milled with the S#2 strategy, which symmetrically removed material from both sides of the plate, in the longitudinal rolling direction. However, the position of the face cutter axis during the machining process was observed to have a significant effect on the surface roughness. A higher surface roughness was measured in the areas of the tool point transition from the up-milling direction to the down-milling direction (tool axis path) for all analysed strategies (*Ra* = 0.63–0.68 µm). The best values were obtained for the up-milling direction, but in the area of the smooth execution of the process (*Ra* = 0.26–0.29 µm), not in the area of the blade entry into the material. A similar relationship was obtained for analysed medians of the arithmetic mean height (*Sa*) and the root-mean-square height (*Sq*). However, in the case of the S#3 strategy, the spreads of results were the lowest.

## 1. Introduction

In the era of the global energy crisis [1], lightweight alloys are being used to manufacture elements for various types of vehicles. These alloys are characterised by a high strength-to-weight ratio due to their low density and reducing energy consumption (electric or fuel) during the exploitation of vehicles manufactured of these alloys [2]. The lightweight alloys often are represented by aluminium alloys, which are used in manufacturing process elements for automotive [2,3], aerospace [4,5] and rail [3]. The main range of components manufactured from thin sheets of aluminium alloys, which require a high proportion of milling in the manufacturing process, are front panels for control systems, housing parts for electrical and electronic components. For these components, both dimensional and surface quality are important aspects of production, especially if the machined surface is also intended to serve as a heat or/and radiation shield. In the manufacturing process of aluminium alloy components, blank (semi-finished) products with small machining allowances are often used to reduce costs [6,7]. This is largely made possible by the cold rolling process used in manufacturing metallurgical materials from aluminium alloys, which allows obtaining fairly low and stable dimensional tolerances [8]. An additional solution for cost reduction is the application of only single fixing while machining. Unfortunately, these solutions carry the risk of a significant effect of residual stresses remaining after the cold rolling process on the results of the machining process [9,10,11]. The cold rolling process introduces anisotropic properties by deforming plastically the processed material. This anisotropy significantly affects the mechanical properties of aluminium alloy sheets [12]. Although the cold rolling process is often followed by stress-relieving processes, there are still residual stresses at the sheet surface [13,14]. Residual stresses occur on both sides of the sheet, which creates a certain balance to ensure the right shape and dimensions. Hattori et al. [15] showed that residual stresses after the cold rolling process of aluminium alloy occur up to a depth of about 1 mm and reach up to 50 MPa. In the case of thin sheets (up to 12 mm thick), this constitutes a significant part of the whole thickness. Therefore, the removal of material with residual stresses from one side of the sheet may lead to deformation of the element due to the residual stresses on the surface of the other side of the sheet. This phenomenon was observed by Dobrzynski et al. [16] while analysing flatness after the face milling process with different strategies. The residual stress distributions created after plastic deformation can be predicted by numerical models or algorithms presented in works by Ding et al. [17], Mutafi et al. [18] and Chen et al. [19]. Sedlak et al. [20] showed that residual stresses are also created after the face milling process. Dobrzynski et al. [16] showed that strategies of removal of material while face milling of cold-rolling thin plates have a significant effect on flatness deviations. The previous work [21] showed that the direction of paths of the face milling process in relation to the cold rolling direction of aluminium alloy plates, considering the anisotropy of the rolled material, also affects the flatness of machined surfaces. Pimenov et al. [22] in their work proposed a mathematical model for determining the deviation from flatness, taking into account the parameters of milling and tool wear. The successful experience of monitoring flatness deviation, depending on the flank wear of the cutter and the engine power of the machine using an artificial, tool is shown in [23]. Additionally, Nowakowski et al. [24] noted in their work that the strategy of the face milling process also affects the heat flux at the tool–workpiece interface, which determines the temperature in the entire thermodynamic system. 

In addition to flatness, surface roughness is another important parameter for the determination of the quality of the machined surface that is most often assessed. Often, the surface roughness of a mechanical component determines its functionality in the range of its intended use [25,26]. The values of surface roughness parameters mainly depend on the cutting process parameters [27] and the cutting edge geometry, which is taken into account by models predicting the values of these parameters [28,29,30]. The surface roughness also depends on the relative position of the face milling tool towards the workpiece [31]. The quality of the machined surface may also depend on the wear level of the cutting edge [32,33]. Cutting edge wear during the cutting process and general cutting process can be assessed by monitoring cutting forces [34], whereas the monitored cutting forces can be used to predict the quality of the machined surface [35]. A lot of research works have been devoted to the analysis and optimisation of cutting process parameters to achieve low surface roughness levels [36,37,38], high dimensional accuracy [39], high process efficiency [40,41] and reduced energy consumption during the machining process [42]. Many previous works have also been devoted to different strategies for implementing cooling and lubrication of the cutting process of aluminium alloys and their effect on the surface quality after machining [43,44]. Maruda et al. [45] presented a study on the effect of minimum quantity lubrication and surface roughness in the turning process. Jebaraj et al. [46] studied the effect of cryogenic CO_2_ and LN_2_ coolants on the milling of aluminium alloy and found that the conventional fluid coolant offers a better surface roughness value (*Ra*) over cryogenic coolants. Gupta et al. [47] analysed a hybrid method that included a cooling process by nitrogen and a lubrication process by minimum quantity lubrication. The dynamic effects of the face milling process on surface roughness were also analysed [48]. Analysis of the open literature shows that many previous scientific works have been devoted to the effect of cutting process parameters, lubrication method, dynamics of the cutting process system and tool path execution strategy on surface roughness. However, the effect of the strategy of removing the outer layers of the material manufactured by the cold rolling process on surface roughness has not been analysed. Work presented by Robinson et al. [13] and Hattori et al. [15] has shown that residual stresses are contained in the outer layers of aluminium alloys produced by cold rolling. Dobrzynski et al. [16] showed that strategies for removing the outer layers of cold-rolled material significantly affect flatness deviations. It is supposed that the resulting flatness disturbances during the face milling process may influence the values of geometrical parameters of the cutting process, e.g., depth of cut. Additionally, with the occurrence of dynamic variations in the depth of cut during the process, this influence can be significant. 

The aim of this work was to analyse the effect of the strategy of removing the outer layers of the material manufactured with the cold rolling process on surface roughness, taking into account the rolling direction.

## 2. Materials and Methods

### 2.1. Materials

Plates of EN AW-6082-T6 alloy (according to the standard EN 485 [49]) were used in the investigation. The basic mechanical properties of the tested material were as follows: yield stress *R*_p0.2_ = 260 MPa, tensile strength *R*_m_ = 310 MPa, modulus of elasticity = 70 GPa, hardness = 95 HV, and elongation at break *A*_5_ = 10%. The chemical composition is shown in Table 1.

**Figure 1:**
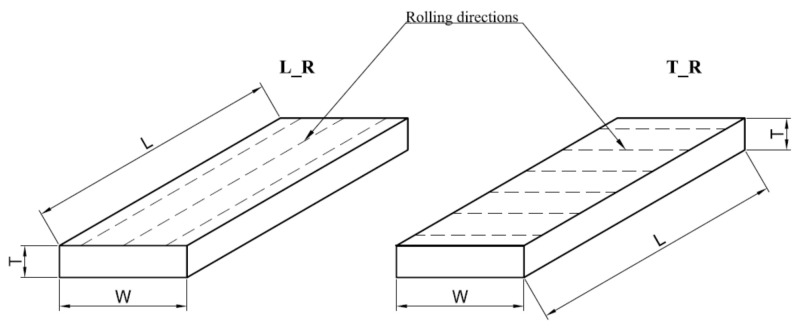
Rolling directions for tested aluminium alloy samples. L_R—longitudinal rolling direction; T_R—transverse rolling direction; L—length of the sample (mm); W—width of the sample (mm); T—thickness of the sample (mm).

Investigated plates were manufactured using the cold rolling process. Samples for experimental testing were prepared with three thickness dimensions: T = 6, 8 and 12 mm. The sheets with nominal dimensions (1000 mm × 2000 mm) were cut on rectangular samples with dimensions W = 60 mm × L = 200 mm for each thickness. The cutting process was carried out on the water jet cutting machine MAXIEM 1530 (OMAX Corporation, Kent, WA, USA). This cutting method of the metal material ensures good dimensional quality and does not introduce structural changes in the material caused by temperature. The structural changes could occur during laser or plasma cutting. Circular saw cutting would require additional processing to ensure the required parallelism of the sides of the samples and surface accuracy, which are necessary for proper clamping in a vice. Samples of any thickness were prepared in two versions: the first one with the direction of cold rolling along the longer side (L_R) and the second one with the direction of cold rolling perpendicular to the longer side (T_R) (Figure 1).

### 2.2. Machine Tool and Cutting Tool

**Figure 2:**
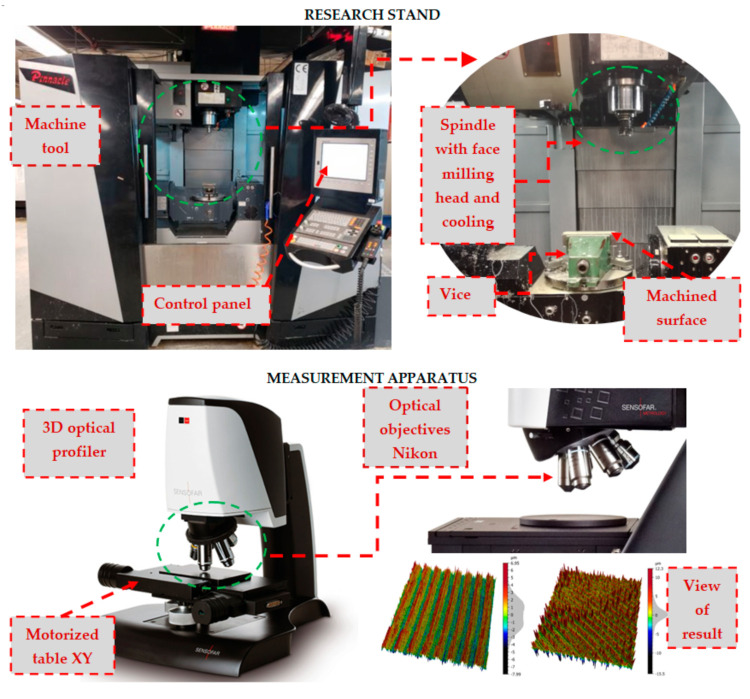
Schematic diagram of the experimental arrangement.

The face milling process of samples was carried out on the multi-axis milling centre AX320 Pinnacle (Pinnacle Machine Tool Co., Ltd., Taichung City, Taiwan). The milling process was performed by the machine tool in accordance with the CNC programme on the Heidenhain TNC 640 control system (Figure 2) (TNC 640, 340590-04, 2014, DR. JOHANNES HEIDENHAIN GmbH, Traunreut, Germany). Samples were mounted using the standard vice with a jaws length of 100 mm. The samples were clamped in the jaws of the vice to a depth of 3 mm. The samples were supported from the bottom with steel plates. These steel plates supplemented the clamping set of tested samples on the machine table, which was in accordance with the practice of the elementary engineer.

A face milling head equipped with 5 cutting tool inserts type APMT 160408 grade of cemented carbide NA20, N20 group of the application according to ISO 513 [51] (Derek Tools Co., Ltd., Yinzhou District, Ningbo, China), which is recommended for the machining of aluminium alloys, was used in experimental tests. The basic dimensions of the cutting tool and cutting edge are shown in Table 2. The dimension of the cutter diameter (Table 2) allowed full-width processing of tested plates in one working path of the tool. The width of the cut was a_e_ = 60 mm. The kinematic parameters of the face milling process used during the experimental tests are shown in Table 3. Many of the previous research works have shown that cutting process parameters have a significant effect on surface roughness [27,34,42]. The feed per tooth has a significant effect on the cutting process [38] and on surface roughness [27,34,36,42,46]; however, the cutting speed and depth of cut also significantly affect the surface quality, as shown in the works [36,42,52]. To limit the effect of cutting parameters on the results of analyses of the effects of face milling strategies on the surface roughness, one set of cutting process parameters was used in the experimental study. The applied cutting parameters were selected based on the industrial machining processes that were the inspiration for conducting experimental tests. An external tool cooling system integrated with the milling centre AX320 was used during machining tests (Figure 2). The machining fluid Blasocut 2000 Universal (Blaser Swisslube AG, Hasle-Rüegsau, Switzerland) was used during experimental tests.

### 2.3. Face Milling Strategies

**Figure 3:**
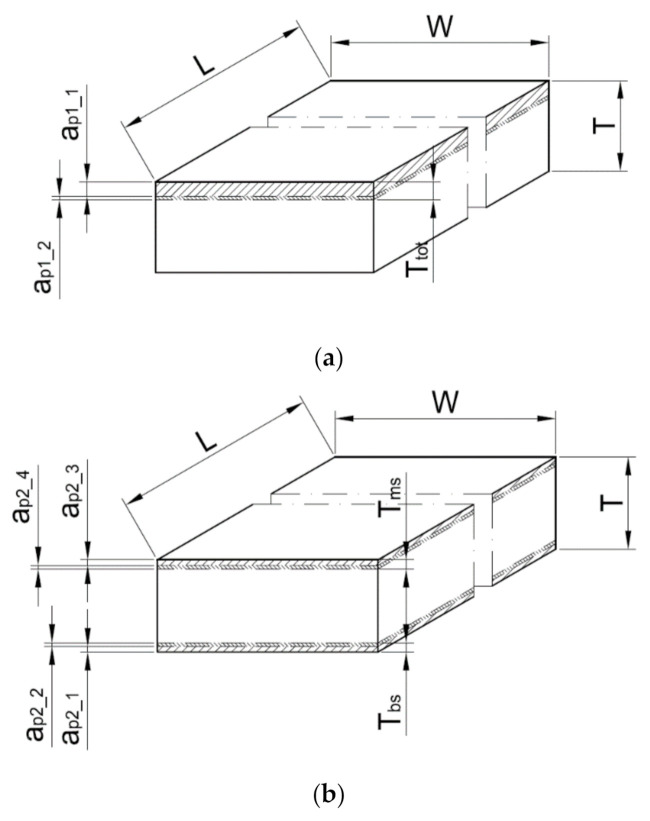

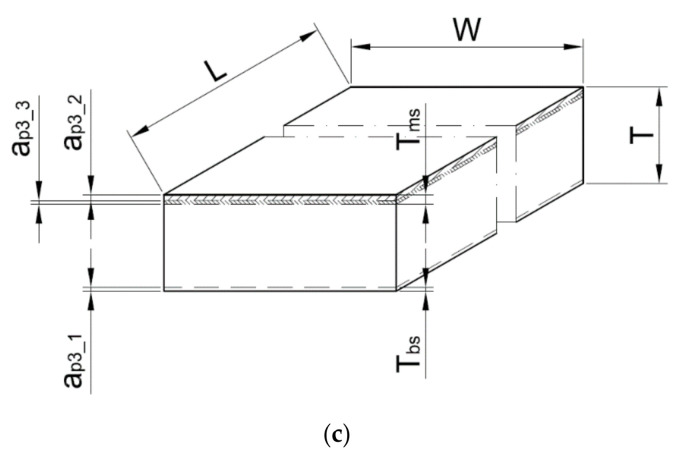
Three experimental research strategies for face milling of aluminium alloy plates: strategy S#1 (**a**), strategy S#2 (**b**) and strategy S#3 (**c**). Where: L—length of the sample (mm); W—width of the sample (mm); T—thickness of the sample (mm), *T_tot_*, *T_bs_*, *T_ms_*—thicknesses of material removed (mm); *a_p_*_1_1_, *a_p_*_1_2_, *a_p_*_2_1_, *a_p_*_2_2_, *a_p_*_2_3_, *a_p_*_2_4_, *a_p_*_3_1_, *a_p_*_3_2_, *a_p_*_3_3_ —depths of cut (mm).

The face milling experimental tests consisted of removing a layer of material with a total thickness of *T*_tot_ = 1 mm from the aluminium alloy plates. This value was selected according to results obtained by Hattori et al. [15]. The removing process was carried out with the use of three strategies of machining. The types of strategies were selected based on experience and industry reports on the effectiveness of the analysed strategies in the reduction in flatness deviations. The first strategy, S#1, was expected to remove the total thickness of the material that was provided only from one side of the plate—the main side (*T*_tot_ = *T*_ms_). The milling process in strategy S#1 was carried out in two steps with the use of two different depth of cut values (*a*_p1_1_ and *a*_p1_2_) (Figure 3). Strategy S#2 included machining from both sides of the plates. In this case, the layer thicknesses were symmetrical (*T*_ms_ = *T*_bs_), and on each side, the layer thickness was removed by two work movements with the depth of cut, *a*_p2_. Firstly, the layer of the back of the plate (*T*_bs_) was machined. The last strategy (S#3) consisted of machining both sides, but firstly, the thin layer was removed from the back by one working movement (*T*_bs_ = *a*_p3_1_). The main side was machined in work movements applied with two different depths of cut, *a*_p3_2_ and *a*_p3_3_. All strategies are shown in Figure 3, and depths of cut for all strategies are posted in Table 4.

### 2.4. Measurement Methodology of Surface Roughness of the Main Side of Plates

The 3D Optical Profiler S neox (Sensofar, Terrassa, Spain) with objective 5× EPI v35 (Nikon, Tokyo, Japan) was used for surface topography measurements of the analysed workpieces (Figure 2). During the investigation, the measuring system was controlled by SensoSCAN 6.6 software (2019, Sensofar, Terrassa, Spain) and the surface analysis was carried out using MountainsMap 7.1 software (2019, Digital Surf Headquarters, Besançon, France). The basic details of the measurement were set as follows:

- Topography: 1353 × 23,632 pixels
- Pixel size: 2.6 µm/pixel
- Z-Scan step of 12 μm
- Threshold 3%
- Algorithm: Confocal Fusion

The selected parameters do not comply with the recommended parameters for this type of measurement [53,54]. However, their selection made it possible to carry out the analysis over the entire width of the samples and also to show the differences in the obtained values of surface roughness with different strategies used. All measurements were carried out under identical conditions (temperature, lighting, operator, etc.).

**Figure 4:**
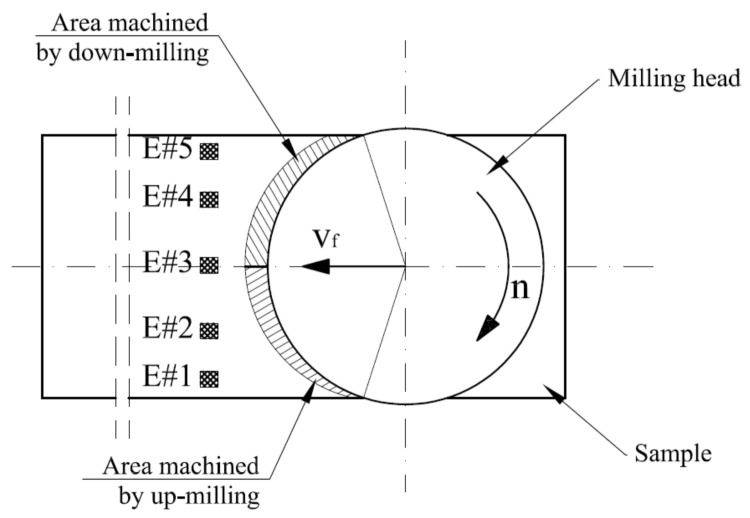
Location of surface topography measurement areas on the sample width, where E#1, E#2, E#3, E#4 and E#5 are names of measurement areas; *v_f_*—feed velocity (mm·min^−1^); *n*—rotational speed (min^−1^).

The positions of the extracted areas 3.49 mm × 4.00 mm (1353 points × 1551 points) were set to cover the entire width of the sample. The distance of the beginning of the area from the edge of the sample was established at 2.5 mm (E#1), 13.5 mm (E#2), 28.5 mm (E#3), 43.5 mm (E#4) and 54.5 mm (E#5) (Figure 4). For such extracted area’s median, spatial filtering with spatial masks (also called window, filter, kernel), 3 × 3 and 9 × 9 sizes, were engaged. The size of the masks determines the number of neighbouring pixels that influence the output value. These filters reduced the noise on the investigated samples as well as the micro-roughness of analysed surfaces. The filter replaces a point by the median only if the point’s Z-value is in the indicated range of the neighbours’ Z-values. This means that the value is not modified if it is close to its neighbours’ values. Additionally, to analyse surface texture, the general slope of a sample using the levelling process was removed. The Level operator was applied, in accordance with ISO 25178 [55], which is based on the least-squares (LS) form-fitting such as levelling using an LS-plane. 

The analysis of the extracted areas under investigation was based on the roughness of the surfaces. These LS surfaces were obtained by applying a Gaussian filter, in accordance with ISO 16610-61 [56]. The choice of the nesting index of 0.8 mm related to obtaining a three-dimensional surface texture for defining irregular surface features after the milling process. In addition, the surface was converted into a series parallel to the direction of feed motion profiles, at fixed distances. This series contained 156 profiles of 1353 points. Based on it, selected R-parameters were collected for the 0.8 mm cut-off value using a Gaussian filter (ISO 4287 [57]). 

### 2.5. Mathematical Models for Prediction of the Surface Roughness

The obtained results of the 2D surface roughness parameter *Ra* were compared with the values obtained based on two mathematical models presented in the literature [28,29,30,58]. The first model (Model #1) is popular, often used for prediction of surface roughness in scientific analysis and industry processes, and was proposed by Boothroyd and Knight [28] (Equation (1)).

(1) $$Ra=\frac{0.0321\times f_{z}^{2}}{r_{\varepsilon }}$$

where *f*_z_ is the feed per tooth and *r*_ε_ is the corner radius. 

**Figure 5:**
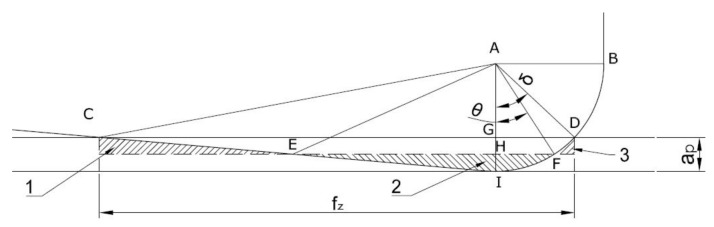
Sketch of one machine mark produced by a small feed per tooth. The dashed line was defined as the mean line, which made area 1 plus area 3 equal to area 2. Where: *a_p_*—depth of cut (mm), *f_z_*—feed per tooth (mm), δ and *θ*—are the angles (°).

The second, newest model (Model #2) is represented by Equation (2). This model was proposed by Wang et al. [30] for a face milling process with triangular inserts. The results presented for this model showed better accuracy of the predicted values, especially for processes with higher feed per tooth [30]. Wang et al. [30] presented in their work a three prediction models related to the proportion between the corner radius of the cutting insert and the feed per tooth. In our analysis, the model proposed for small feed-per-tooth values and for the relation *f_z_* < *r_ε_* was used (Figure 5).

(2) $$Ra=\frac{2r_{\varepsilon }^{2}}{f_{z}}\cdot \left(\theta -\sin\theta \cdot \cos\theta \right)$$

where *θ* is the angle between the lowest point and the point intersecting the mean line and round profile and can be calculated as

(3) $$\theta =\arccos\left(\frac{r_{\varepsilon }}{f_{z}}\cdot \delta +\frac{1}{2}\cos\delta \right)$$

and *δ* is the angle between the lowest point and the intersection point between two continuous machine marks and can be calculated using the following equation:

(4) $$\delta =\arcsin\left(\frac{f_{z}}{2r_{\varepsilon }}\right)$$

## 3. Results and Discussion 

**Figure 6:**
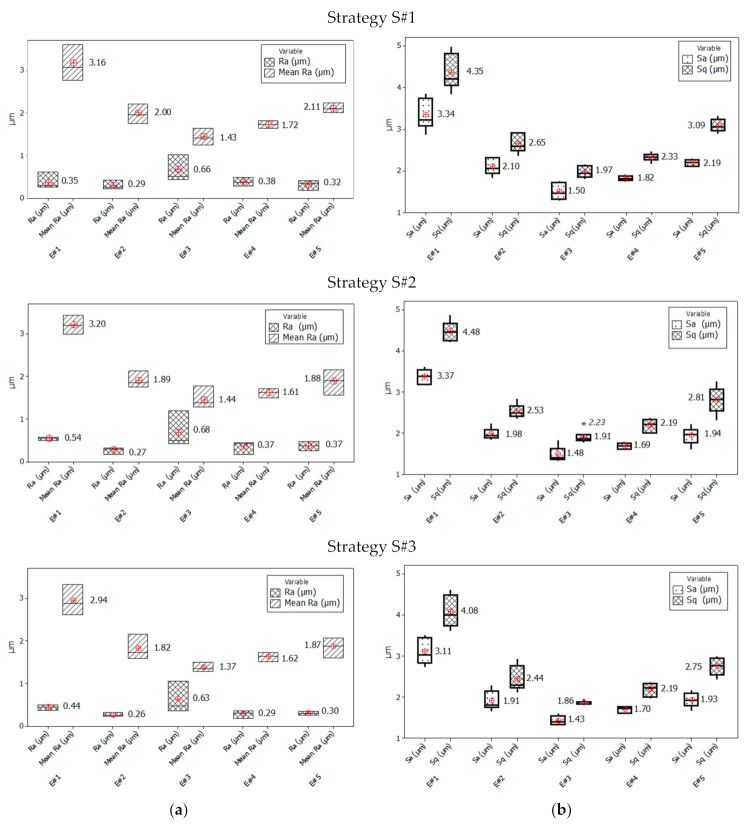
Centre and spread of 2D (**a**) and 3D (**b**) surface roughness parameters for three milling strategies: S#1, S#2 and S#3. 2D parameters (**a**): *Ra* for the mean *R* profile and mean *Ra* parameter. 3D parameters (**b**): *Sq* and *Sa* parameters. The box plot presents the mean, the median, the interquartile range box and the range of the data. E#1, E#2, E#3, E#4 and E#5 are names of measurement areas. * Outlier value *Sq* = 2.23 μm, in 3D (**b**).

The obtained results of the surface quality after the face milling process, using three cutting strategies, allowed analysing the effect of the applied strategy and the differentiation of surface roughness parameters on the sample width (*W)*. In the central zone of the sample (E#3), in the area of the transition of the cutting edge from up-milling to down-milling (Figure 4), for each applied cutting strategy (Figure 2), the mean parameters surface roughness *Ra* was the lowest (Figure 6a). On these areas (E#3), the values of the mean surface roughness parameter *Ra* were in the range of 1.24–1.77 µm. The *Ra* values for the mean profile *R* were in the range of 0.35–1.20 µm (Figure 6a). A similar relationship was obtained for the analysed medians of arithmetic mean height (*Sa*) and root-mean-square height (*Sq*) [53,54]. In the centre part of the sample (E#3), the values of *Sa* and *Sq* were at similar levels for each analysed face milling strategy (Figure 6b). However, in the case of the S#3 strategy, the spreads of results were the lowest (Figure 6b). In the up-milling areas (E#1), the mean values of the surface roughness parameters were twice as high in relation to centre zones (#E3) and were as follows: *Ra* = 2.62–3.60 µm, *Sa* = 3.04–3.38 µm, and *Sq* = 4.01–4.45 µm (Figure 6). The smallest median values of the mean 3D roughness parameters (*Sa* and *Sq*) for the up-milling areas (E#1 and E#2) were obtained for the machining process using the S#3 strategy. However, these values had a relatively large spread of results. The smallest spread of 3D parameters in the up-milling areas was observed for strategy S#2, while for 2D parameters (*Ra*), the smallest spread was recorded for strategy S#3. 

**Figure 7:**
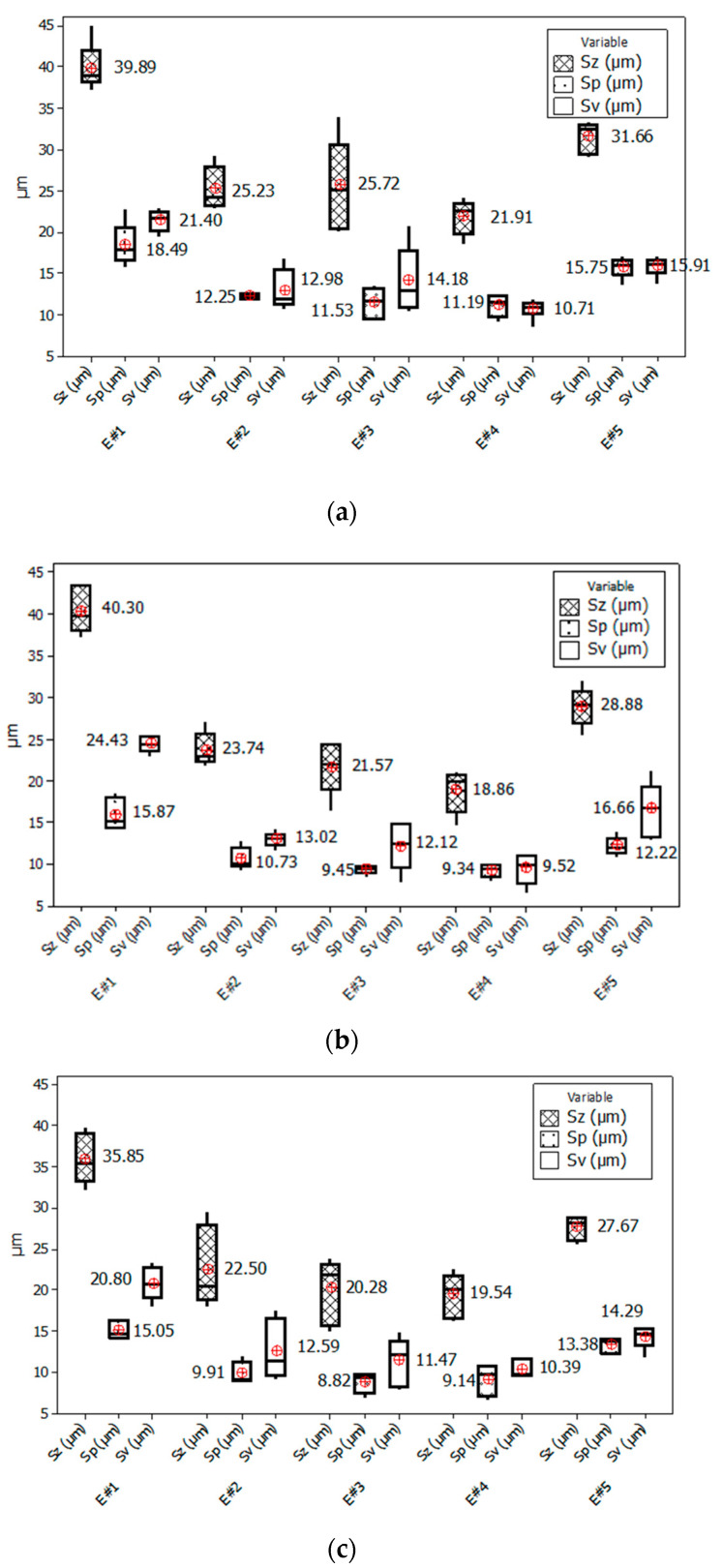
Centre and spread of *Sz*, *Sp* and *Sv* parameters for three milling strategies: S#1 (**a**), S#2 (**b**) and S#3 (**c**). The box plot presents the mean, the median, the interquartile range box and the range of the data. E#1, E#2, E#3, E#4 and E#5 are names of measurement areas.

The mean values of surface roughness parameters, obtained for the areas where the milling cutter performed down-milling, were slightly higher than for the central areas. The mean values of 2D parameters (*Ra*) were in the range of 1.50–2.24 µm for tested strategies (Figure 7a), while the 3D parameters were in range of *Sa* = 1.70–2.20 µm and *Sq* = 2.22–3.06 µm (Figure 7b). Additionally, these values were characterised by a noticeably less spread than for the up-milling areas. Interestingly, the lowest spreads in the down-milling areas (E#4 and E#5) of both 2D and 3D parameters were obtained for the S#1 strategy (Figure 6). To better understand the phenomena occurring during the analysed face milling processes, other 3D surface roughness parameters were also investigated, namely *Sz* (maximum height), *Sp* (maximum peak height) and *Sv* (maximum pit height) [53,54] (Figure 7). 

The medians of the *Sz* parameter in areas E#2 to E#4 were at a similar level for each used strategy, around 21 µm in the case of S#2 and S#3 strategies and around 24 µm for the S#1 strategy (Figure 7). However, the spreads of results were smaller for the areas obtained during the down-milling movement of the cutter. 

**Figure 8:**
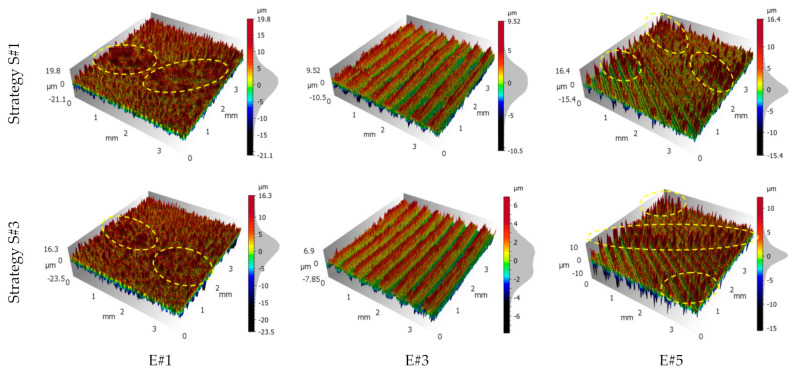
Surface texture views for a 6-mm-thick sample and S#1 and S#3 milling strategies. E#1, E#3 and E#5 are names of measurement areas. The ellipses drawn with a dashed yellow line indicate much higher, unevenly distributed peaks than the general structure.

The highest median value of the *Sz* parameter of nearly 40 µm was obtained at the beginning of the up-milling operation for area E#1 for S#1 and S#2 strategies. This phenomenon is probably caused by the fact that these areas are also the beginning areas for the whole cutting process by individual blades. The application of the S#3 strategy allowed obtaining better results by nearly 5 µm (*Sz* = 35.32 µm). Analysing the *Sp* and *Sv* parameters, it can be observed that the distribution of heights (represented by the *Sp* parameter) and valleys (represented by the *Sv* parameter) in the case of the S#1 strategy was the most homogeneous (Figure 7). As a result of the S#2 strategy, a predominance of valleys over heights was obtained for the areas milled by the up-milling movement (E#1) of the cutter. In the case of the S#3 strategy, this phenomenon also occurred, however, with less intensity. Additionally, in the case of the S#3 strategy used for areas milled by the down-milling movement (E#5) of the cutter, the spread of the results was small (Figure 7). In Figure 8, examples of the surface texture are shown. These sample surface textures satisfactorily illustrated the results presented in Figure 6 and Figure 7. The proportions between peaks and valleys are noticeable. The uniform distribution in transition areas E#3; the predominance of valleys in areas machined with up-milling motion, E#1; and the intermediate texture in the case of areas machined with down-milling motion, E#5, are visible.

**Figure 9:**
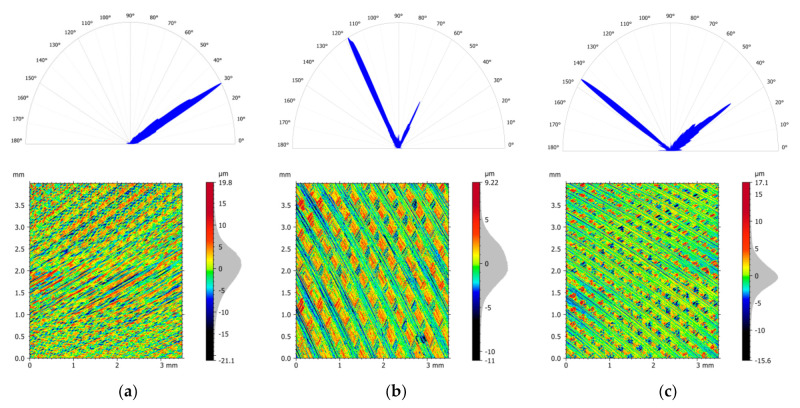
Orientation plot and the view of the surface texture for 6-mm-sample milled using strategy S#1 (*T_R* rolling) and areas E#1 (**a**), E#4 (**b**) and E#5 (**c**).

The direction of the texture grooves in the tested specimens varied in different areas, but these were characteristic of face milling at a given feed velocity, the number of teeth, the tool diameter and the work path position (Figure 9 and Table 5). In the central part of the samples (area E#3), the direction of the texture grooves was around 90° and the angles obtained were in the range of 88.5–91.5° (for isotropy 2.93–3.99%). In 1/3 (E#2) and 2/3 (E#4) of the sample width, the dominant directions were 60° and 120° (with varying levels of isotropy). In 1/3 of the specimen width (E#2), the angles for the first direction (60°) and second direction (120°) were approximately the same in all tested cases. The first entry of the blade in contact with the material produced deeper grooves on the workpiece surface. However, in 2/3 (E#4) of the specimen width in all tested strategies for 6-mm-thick specimens, the opposite situation occurred and the dominant grooves were generated on the surface by the next pass of the milling head blades (first direction 120° and second direction 60°) (Figure 9b). This could be related to the relatively small thickness of the specimen, which affected its stiffness when removing surface residual stresses left over from the cold rolling process [12,15]. This phenomenon occurred on the side of the movable jaw of the vice, which could be due to the fact that during the removal of the layer with residual stresses, the sample with a small thickness somehow deformed elastically during the milling process. On the second side of the samples, which were clamped by the fixed jaw (E#1), the texture grooves formed by the next pass of the cutting edges were practically invisible (Figure 9a).

On the side clamped by the movable jaw in the areas close to the edge of the samples (E#5), both directions of 30° (first blade passage) and 146° (subsequent blade passage) were present. The dominant texture grooves after the next blade pass (similarly as for the E#4 areas) were obtained for the thinnest samples (6 mm) for strategy S#1 and both directions rolled (Figure 9c). A similar phenomenon was obtained for the samples with a thickness of 8 mm milled by strategy S#1 (semi-finished product rolled in the L_R direction), but in this case, the percentage of isotropy was at a relatively high level (44.6%) (Table 5). This situation was also observed for strategies S#2 and S#3 for samples rolled in the T_R direction.

## 4. Comparison of Mathematical Models and Experimental Surface Roughness

The results of the predicted values of the surface roughness parameter *Ra* obtained using two models taken from the literature [28,30] and their comparison with the values obtained from the experiment carried out are shown in Table 6. Both models predicted the same values of the *Ra* parameter, which are lower than the experimental values. The difference between the values obtained from mathematical models and the experiment is noticeable but not significant because the experimental values (average) also have large values of standard deviation. The lowest values of the *Ra* parameter, and, at the same time, closest to the values predicted using mathematical models, were obtained for the S#3 strategy. However, the differences between the average values for individual strategies are not significant, which was observed in the case for the surface flatness and was presented by Dobrzynski et al. [16]. Therefore, it can be concluded that the surface roughness is not affected by the residual stress on the outer surfaces of the material, which is a residue from the cold rolling process. Nevertheless, it is noteworthy that the surface roughness is noticeably affected by the thickness of the face-milled material. For each strategy, the highest, i.e., worst in terms of machining quality, values were obtained for the thinnest samples with a thickness of 6 mm. The mathematical models analysed do not take into account the stiffness of the workpiece material, which in the studied case is represented by the thickness of the workpiece material. Nor do they take into account the position of the cutting tool axis relative to the machined workpiece surface. The effect of this parameter is presented in Figure 6 and Figure 7, as well as in Table 5. In the case of the *Ra* parameter (Figure 6a), the least favourable values were obtained in the path of the working motion of the cutting tool axis. These values are twice as high as those obtained on the surfaces machined by both sides of the cutting tool, for both up- and down-milling motion.

These newly observed phenomena present directions for further research to develop a surface roughness prediction model for the face milling process that takes into account the type and stiffness of the machined material and the position of the tool axis relative to the machined surface.

## 5. Conclusions

In this experimental research, strategies of material removal by the face milling process from aluminium plates manufactured with the cold rolling process and their effects on surface roughness were investigated. The analysed milling strategies take into account the depth of the material with included residual stresses after cold rolling based on literature sources. Based on the experimental tests carried out and analysis the of obtained results, the following can be concluded:

- The cold rolling direction of tested thin aluminium alloy plates does not affect the roughness of face-milled surfaces. The lowest values of *Ra* = 0.34 µm were calculated from a mean profile for the S#3 strategy for an 8-mm-thick plate in the transverse rolling direction. For the longitudinal rolling direction, the same plate thickness and almost the same value of *Ra* = 0.35 µm were calculated from a mean profile.
- The thickness of the face-milled aluminium alloy plates did not significantly affect the 2D and 3D parameters of surface roughness. The highest values of surface roughness 2D parameters were measured for a 6-mm-thick plate milled with the S#2 strategy, *Ra* = 0.46 µm for the transverse rolling direction and *Ra* = 0.48 µm for the longitudinal rolling direction.
- No effect of the strategy of removal of material layers from both sides of the plate, which may contain residual stresses after cold rolling, on the values of surface roughness parameters was observed. The lowest values of 2D surface roughness parameters were obtained for strategy S#3, *Ra* = 0.34 ± 0.07 µm, and the highest values for strategy S#2, *Ra* = 0.48 ± 0.44 µm.
- The phenomenon of the face milling cutter axis position in relation to the machined area effect on surface roughness parameters was observed. The worst values of 2D surface roughness parameters were measured in the areas of the blade transition from the up-milling direction to the down-milling direction (tool axis path) for all analysed strategies (*Ra* = 0.63–0.68 µm). The best values were obtained for the up-milling direction but in the area of the smooth execution of the process (*Ra* = 0.26–0.29 µm). In the up-milling areas, which were the areas of the blade entry into the material (E#1), the mean values of the surface roughness parameters were twice as high in relation to centre zones (#E3) and were as follows: *Ra* = 2.62–3.60 µm, *Sa* = 3.04–3.38 µm, and *Sq* = 4.01–4.45 µm.

## Figures and Tables

**Table 1:** Chemical composition of the material EN AW-6082-T6 according to EN-573-3:2019 [50].

Name of Component Elements	Value	Content
Aluminium, Al	95.2–98.3	%
Chromium, Cr	≤0.25	%
Copper, Cu	≤0.10	%
Iron, Fe	≤0.50	%
Magnesium, Mg	0.6–1.2	%
Manganese, Mn	0.4–1.0	%
Silicon, Si	0.7–1.3	%
Titanium, Ti	≤0.10	%
Zinc, Zn	≤0.20	%
Other, total	≤0.15	%

**Table 2:** Basic dimensions of the cutting tool and cutting edge.

Name of Dimension	Value	Unit
Diameter of milling head, *D*	63	mm
Number of teeth, *z*	5	-
Corner radius, *r*_ε_	0.8	mm
Tool rake angle, *γ*_o_	10°	degrees
Tool minor rake angle, *γ*_o_′	10°	degrees
Tool clearance angle, *α*_o_	11°	degrees
Tool minor clearance angle, *α*_o_′	15°	degrees
Tool cutting edge angle, *κ*_r_	90°	degrees
Tool minor cutting edge angle, *κ*_r_′	5°	degrees

**Table 3:** Kinematic parameters of the face milling process.

Name of Dimension	Value	Unit
Rotational speed, *n*	1400	min^−1^
Cutting speed, *v_c_*	264	m·min^−1^
Feed velocity, *v_f_*	600	m·min^−1^
Feed per tooth, *f*_z_	0.086	mm

**Table 4:** Depths of cut for investigated strategies.

Name of Strategy	Machining Side of Plates	Value of Layer Thickness (mm)	Depth-of-Cut Symbol	Depth-of-Cut Value (mm)
Strategy S#1	Main side, *T*_ms_	1.00	*a_p_*_1_1_	0.75
			*a_p_*_1_2_	0.25
Strategy S#2	Back side, *T*_bs_	0.50	*a_p_*_2_1_	0.25
			*a_p_*_2_2_	0.25
	Main side, *T*_ms_	0.50	*a_p_*_2_3_	0.25
			*a_p_*_2_4_	0.25
Strategy S#3	Back side, *T*_bs_	0.25	*a_p_*_3_1_	0.25
	Main side, *T*_ms_	0.75	*a_p_*_3_2_	0.50
			*a_p_*_3_3_	0.25

**Table 5:** Isotropy and directional properties of surface features.

Sample Thickness		6	6	8	8	12	12	Max.	Min.
Rolling Strategy		T_R	L_R	T_R	L_R	T_R	L_R		
Strategy S#1									
E#1	Isotropy	9.25	9.41	14.1	15.4	18.7	13.2	18.7	9.25
	First direction	29.8°	31.5°	29.5°	29.8°	31.5°	31.7°	31.7°	29.5°
	Second direction	38.2°	38.2°	39.2°	41.3°	39.5°	44.5°	44.5°	38.2°
E#2	Isotropy	3.32	6.64	25.7	9.68	27.6	4.67	27.6	3.32
	First direction	59.5°	58.3°	58.7°	59.5°	60.5°	60.3°	60.5°	58.3°
	Second direction	121°	119°	120°	120°	121°	121°	121°	119°
E#3	Isotropy	2.94	3.18	3.82	3.76	3.82	3.32	3.82	2.94
	First direction	89.5°	88.5°	89°	89°	91°	90.7°	91°	88.5°
E#4	Isotropy	9.59	10.1	33.7	44.2	28.2	24.4	44.2	9.59
	First direction	118°	118°	60.5°	61.7°	61.5°	61.3°	118°	60.5°
	Second direction	61.5°	61.2°	118°	118°	120°	119°	120°	61.2°
E#5	Isotropy	23.7	27.9	29.6	44.6	23	24.4	44.6	23
	First direction	146°	145°	33°	145°	33.8°	33°	146°	33°
	Second direction	33.5°	33°	145°	34°	148°	148°	148°	33°
Strategy S#2									
E#1	Isotropy	17.1	19	7.82	15.1	17.3	7.13	19	7.13
	First direction	29.8°	30.7°	30°	29.8°	31.8°	31.5°	31.8°	29.8°
	Second direction	21.5°	38.7°	22.3°	38.7°	38.2°	38°	38.7°	21.5°
E#2	Isotropy	4.74	6.17	6.47	6.79	4.69	4.8	6.79	4.69
	First direction	59.3°	60°	60°	59.3°	60.5°	60°	60.5°	59.3°
	Second direction	133°	121°	120°	119°	120°	121°	133°	119°
E#3	Isotropy	2.93	3.33	3.32	3.99	3.47	3.35	3.99	2.93
	First direction	89°	90.2°	91.2°	90.7°	90.8°	91°	91.2°	89°
E#4	Isotropy	2.55	25.9	15.5	18.1	15.5	13.7	25.9	2.55
	First direction	119°	119°	62°	61.5°	61.2°	61.3°	119°	61.2°
	Second direction	62°	61.7°	119°	119°	120°	118°	120°	61.7°
E#5	Isotropy	25.5	38.4	38.9	36.5	43.5	38.5	43.5	25.5
	First direction	146°	34°	34.5°	34°	33°	33.3°	146°	33°
	Second direction	34°	146°	146°	146°	147°	147°	147°	34°
Strategy S#3									
E#1	Isotropy	12.8	12.7	26.3	18.7	20.9	20	26.3	12.7
	First direction	32°	31.5°	28.8°	30°	31.5°	31.5°	32°	28.8°
	Second direction	37.7°	41°	38°	22.5°	44.2°	38°	44.2°	22.5°
E#2	Isotropy	2.72	3.02	4.53	9.48	13.1	8.96	13.1	2.72
	First direction	60.5°	60.2°	58.5°	60.2°	60.7°	60.3°	60.7°	58.5°
	Second direction	121°	118°	119°	122°	120°	119°	122°	118°
E#3	Isotropy	3.22	3.32	3.67	3.76	3.59	3.6	3.76	3.22
	First direction	90.5°	90.3°	91.5°	91.5°	91°	91°	91.5°	90.3°
E#4	Isotropy	3.11	10.2	11	13.5	14.1	12.1	14.1	3.11
	First direction	119°	118°	61.8°	62.2°	61°	61.5°	119°	61°
	Second direction	61.5°	61°	118°	120°	120°	120°	120°	61°
E#5	Isotropy	36.2	44.2	34.1	40.8	42.5	37.3	44.2	34.1
	First direction	147°	33.5°	34.7°	34.7°	32.7°	33.5°	147°	32.7°
	Second direction	34.3°	147°	144°	147°	148°	147°	148°	34.3°

**Table 6:** Comparison of mathematical models and experimental surface roughness.

Face Milling Strategy	Sample Thickness (mm)	Rolling Strategy		Model #1	Model #2
		T_R	L_R		
		*Ra* (µm)			
S#1	6	0.44 ± 0.33	0.40 ± 0.32	0.30	0.30
	8	0.36 ± 0.10	0.39 ± 0.10		
	12	0.37 ± 0.10	0.46 ± 0.11		
S#2	6	0.46 ± 0.44	0.48 ± 0.31		
	8	0.46 ± 0.09	0.43 ± 0.10		
	12	0.41 ± 0.10	0.44 ± 0.09		
S#3	6	0.43 ± 0.36	0.45 ± 0.33		
	8	0.34 ± 0.07	0.35 ± 0.10		
	12	0.37 ± 0.10	0.36 ± 0.33		

## Data Availability

Data sharing is not applicable to this article.

## Nomenclature

*A*_5_	elongation at break
*D*	diameter of milling head
E#1, E#2, E#3, E#4, E#5	areas of surface roughness measurement
L	length of samples
L_R	longitudinal cold rolling direction
*Ra*	2D surface roughness parameter
*R*_m_	tensile strength
*R*_p0.2_	yield stress
S#1, S#2, S#3	face milling strategies
*Sa, Sp, Sq, Sv, Sz*	3D surface roughness parameters
T_R	transversal cold rolling direction
T	thickness of samples
*T*_bs_	thickness of material removed from the back of the sample
*T*_ms_	thickness of material removed from the main side of the sample
*T*_tot_	total thickness of material removed from both sides of the sample
W	width of samples
*a_e_*	width of cut
*a*_p1_1_, *a*_p1_2_, *a*_p2_1_, *a*_p2_2_, *a*_p2_3_, *a*_p2_4_, *a*_p3_1_, *a*_p3_2_, *a*_p3_3_	depths of cut for given strategies
*f*_z_	feed per tooth
*n*	rotational speed
*r*_ε_	corner radius
*v*_c_	cutting speed
*v*_f_	feed velocity
*z*	number of teeth
*α*_o_, *α*_o_′	clearance angle and minor clearance angle
*γ*_o_, *γ*_o_′	rake angle and minor rake angle
*κ*_r_, *κ*_r_′	cutting edge angle and minor cutting edge angle
*δ*	angle determined by graphical methods [30]
*θ*	angle determined by graphical methods [30]
